# Vector-borne pathogens of zoonotic concern in dogs from a *Quilombola* community in northeastern Brazil

**DOI:** 10.1007/s00436-022-07661-x

**Published:** 2022-09-14

**Authors:** Lucia Oliveira de Macedo, Marcos Antonio Bezerra-Santos, Carlos Roberto Cruz Ubirajara Filho, Kamila Gaudêncio da Silva Sales, Lucas C. de Sousa-Paula, Lidiane Gomes da Silva, Filipe Dantas-Torres, Rafael Antonio do Nascimento Ramos, Domenico Otranto

**Affiliations:** 1 Graduate Program in Animal Biosciences, Federal Rural University of Pernambuco, Recife, Brazil; 2 Laboratory of Parasitology, Federal University of the Agreste of Pernambuco, Garanhuns, Brazil; 3 Department of Veterinary Medicine, University of Bari, Valenzano, Italy; 4 Laboratory of Immunoparasitology, Department of Immunology, Aggeu Magalhães Institute, Oswaldo Cruz Foundation (Fiocruz), Recife, Brazil; 5 Department of Pathobiology, Faculty of Veterinary Science, Bu-Ali Sina University, Hamedan, Iran

**Keywords:** *Hepatozoon canis*, *Rickettsia felis*, Zoonotic pathogens, Ticks, Fleas, Vectors

## Abstract

Canine vector-borne pathogens (CVBPs) comprise a group of disease agents mainly transmitted by ticks, fleas, mosquitoes and sand flies. In this study, we assessed the presence of CVBPs in an Afro-descendent community (*Quilombola*) of northeastern, Brazil. Dog blood samples (*n* = 201) were collected and analyzed by rapid test for the detection of antibodies against *Leishmania* spp., *Anaplasma* spp., *Ehrlichia* spp. and *Borrelia burgdorferi* sensu lato (s.l.), and antigens of *Dirofilaria immitis*. In addition, polymerase chain reactions were performed for Anaplasmataceae, *Babesia* spp., *Hepatozoon* spp., *Rickettsia* spp. and *B. burgdorferi* s.l. Overall, 66.7% of the dogs scored positive to at least one pathogen at serological and/or molecular methods. Antibodies against *Ehrlichia* spp. were the most frequently detected (57.2%; *n* = 115/201), followed by *Anaplasma* spp. (8.5%; *n* = 17/201), *Leishmania* spp. (8.5%; *n* = 17/201) and *B. burgdorferi* s.l. (0.5%; *n* = 1/201). For *D. immitis*, 11 out of 201 (5.5%) animals scored positive. At the molecular analysis, 10.4% (*n* = 21/201) of the samples scored positive for *Babesia* spp./*Hepatozoon* spp., followed by Anaplasmataceae (5.0%; *n* = 10/201) and *Rickettsia* spp. (3.0%; *n* = 6/201). All samples were negative for *B. burgdorferi* s.l. Our data demonstrated the presence of CVBPs in the studied population, with a high seropositivity for *Ehrlichia* spp. In addition, considering the detection of zoonotic pathogens in dogs and their relationship with people from *Quilombola* communities, effective control strategies are advocated for minimizing the risk of infection in this socially vulnerable human population and their pets.

## Introduction

Canine vector-borne diseases (CVBDs) are caused by a group of pathogens mainly transmitted by ticks (e.g., *Babesia* spp*., Ehrlichia* spp., *Anaplasma* spp., *Rickettsia* spp.), fleas (e.g., *Rickettsia felis*, *Dipylidium caninum*), mosquitoes (e.g., *Dirofilaria* spp.) and sand flies (e.g., *Leishmania* spp.) (Otranto 2018; Maggi and Krämer 2019; Alhassan et al. 2021). The zoonotic potential of some CVBD-causing agents represents a threat to human health, especially in the tropics and subtropics, as the climatic conditions of these areas are conducive to the growth and proliferation of arthropod vectors (Dantas-Torres 2008; Maggi and Krämer 2019). The CVBDs of major veterinary and public health concern are leishmaniasis and heartworm disease (HWD), followed by anaplasmosis, ehrlichiosis and babesiosis (Dantas-Torres et al. 2020; Borges et al. 2021). In Brazil, dogs can be infected by zoonotic *Leishmania* spp., including *Leishmania infantum*, *Leishmania amazonensis* and *Leishmania braziliensis* (Belo et al. 2013; Souza et al. 2019). *Leishmania* spp. infection occurs mostly through the bites of infected female phlebotomine sand flies (Alvar et al. 2012). Leishmaniases are still predominantly rural diseases, with wildlife species (e.g., foxes, opossums and rodents) acting as principal hosts of the parasites (Roque and Jansen 2014; Bezerra-Santos et al. 2021). However, dogs are considered the main reservoirs of *L. infantum* and source of infection to the vectors in both rural and urban areas (Dantas-Torres 2007). Another important parasite of zoonotic potential in Brazil is *Dirofilaria immitis*, the agent of HWD, often characterized by severe cardiopulmonary disorders which may be fatal. The distribution of *D. immitis* overlaps that of mosquito vectors of the genera *Culex*, *Aedes*, *Ochlerotatus* and *Anopheles* (Ahid and Lourenço-Oliveira 1999; Simón et al. 2012; Ramos et al. 2016; Maggi and Krämer 2019; Brianti et al. 2021; Mendoza-Roldan et al., 2021). For example, a previous study demonstrated the presence of competent vectors of *D. immitis* (e.g., *Ochlerotatus scapularis* and *Ochlerotatus taeniorhynchus*) occurring throughout the year in an endemic area of Brazil (Labarthe et al. 1998), with a correlation between the high populations of arthropod vectors and the odds of infection in dogs and humans (Bendas et al. 2019).

*Anaplasma platys*, *Ehrlichia canis* and *Babesia vogeli* are the major causative agents of canine anaplasmosis, ehrlichiosis and babesiosis in Brazil (Dantas-Torres 2008), which are characterized by a variety of clinical signs (e.g., pyrexia, lethargy, weight loss, lymphadenopathy, bleeding tendencies, hemoglobinuria, petechiae, jaundice and even death) (Ybañez et al. 2018) and hematological alterations (e.g., anemia and thrombocytopenia) in infected animals. The main biological vector of these hemoparasites is the brown dog tick (i.e., the recently proposed *Rhipicephalus linneai*, also referred to as the tropical lineage of *Rhipicephalus sanguineus* sensu lato by Šlapeta et al. 2021), which has also been associated with the transmission of other pathogens, such as *Hepatozoon canis* and bacterial species belonging to the spotted fever group rickettsiae (SFGR) (Ramos et al. 2014; Santos et al. 2018; Alhassan et al. 2021). Animals coinfected by multiple CVBD-causing pathogens are at higher risk of developing severe clinical signs (de Caprariis et al. 2011). For example, a study estimated that dogs with leishmaniasis are 12 times more likely to be coinfected with *E. canis* compared to healthy ones, with increased severity of clinical presentation, and potential reduction in the efficacy of the treatment (Attipa et al. 2018).

In Brazil, there are an estimated number of about 3,000 *Quilombola* communities composed by descendants of slaves of African origin, usually located in remote rural areas (Conde et al. 2020). People from these communities commonly live from agriculture and/or use of forest resources (Conde et al. 2020). In these areas wandering dogs, as well as owned restricted and semi-restricted ones are usually part of the domestic fauna. Data on canine zoonotic pathogens from these communities are scarce, with a single report recording a prevalence of 8.4% for *L. infantum* in skin samples from dogs (Silva et al. 2017). Nevertheless, the presence of wandering dogs, vectors and deficient sanitary conditions suggest that other pathogens of veterinary and public health concern may occur in these areas. Thus, the present study was aimed to assess the occurrence of CVBD-causing pathogens in a *Quilombola* community in the northeastern region of Brazil.

## Material and methods

### Study area

This study was conducted in a *Quilombola* community in the municipality of Garanhuns (08°53′25" S, 36°29′34" W; 896 m above sea level), Pernambuco, Northeastern Brazil. Semi-restricted or unrestricted dogs are frequently present in the study area and live in contact with other domestic animals (e.g., cats, birds and domestic ruminants) and wildlife (e.g., opossums, rodents and birds). The study area has a tropical savanna climate with dry-summer characteristics, which corresponds to the Köppen climate classification category “Aw,” presenting a mean annual temperature of 20 ºC (range, 16–30 ºC), relative humidity of 76% (38–100%) and precipitation of 873 mm (751–1000 mm) (Barbosa et al. 2016), with rains concentrated from April to July (Andrade et al. 2008).

### Sample collection and laboratory analysis

From July 2021 to January 2022, blood samples (1.5–5 ml) were collected from cephalic vein of 201 dogs. Samples were divided into two aliquots: one stored in tubes containing EDTA, and the other in tubes without anticoagulant, for molecular and serological analysis, respectively. Data on sex, dog categories (i.e., restricted and semi-restricted according to Otranto et al. 2017), overnight stay and the presence of ectoparasites were recorded for each dog. The serum samples were tested by an immunochromatographic assay (DPP® CVL, BioManguinhos) for anti-*Leishmania* spp. antibodies and by an ELISA rapid test (SNAP® 4Dx Plus, Idexx), which detects antibodies to *Anaplasma* spp. (*A. platys*/*A. phagocytophilum*), *Ehrlichia* spp. (*E. canis*/*E. ewingii*), *B. burgdorferi* s.l. and antigens of *D. immitis*. All tests were performed according to the manufacturer’s instructions.

Genomic DNA was extracted from 200 μl aliquots of EDTA-treated blood samples using GenUP DNA Kit (Biotechrabbit, Berlin, Germany) following the manufacturer’s instructions. All samples were tested for the presence of Anaplasmataceae, *Borrelia* spp., *Rickettsia* spp., *Babesia* spp. and *Hepatozoon* spp. by conventional PCR, using the primers reported in Table 1.

**Table 1:** Primers for the detection of vector-borne pathogens in Brazil

Pathogen	Gene	Amplicon size (bp)	Primer sequences	Reference
*Rickettsia* spp.	*glta*	401	5’GCAAGTATCGGTGAGGATGTAAT3’ 5’GCTTCCTTAAAATTCAATAAATCAGGAT3’	Labruna et al. (2004)
*Rickettsia* spp.	*ompA*	532	5’ATGGCGAATATTTCTCCAAAA3’ 5’AGTGCAGCATTCGCTCCCCCT3’	Regnery et al. (1991)
Anaplasmataceae	16S	345	5’GGTACCYACAGAAGAAGTCC3’ 5’TAGCACTCATCGTTTACAGC3’	Parola et al. (2000)
*Borrelia* spp.	*fla*	482	5’AGAGCAACTTACAGACGAAATTAAT3’ 5’CAAGTCTATTTTGGAAAGCACCTAA3’	Skotarczak et al. (2002)
*Babesia* spp. /*Hepatozoon* spp.	18S rRNA	350–400	5’CCAGCAGCCGCGGTAAATTC3’ 5’CTTTCGCAGTAGTTYGTCTTTAACAAATCT3’	Tabar et al. (2008)

PCR products were purified (Thermo Scientific™ FastAP™ Thermosensitive Alkaline Phosphatase) and sequenced using the same forward and reverse primer sets reported in Table 1 for each pathogen, employing the Big DyeTerminator v.3.1 chemistry in a 3130 Genetic analyzer (Applied Biosystems, California, USA) in an automated sequencer (ABI-PRISM 377). Nucleotide sequences were edited, aligned and analyzed using Mega 7.0 software and compared with reference sequences available on GenBank database using Basic Local Alignment Search Tool (BLAST; http://blast.ncbi.nlm.nih.gov/Blast.cgi).

### Data analysis

Descriptive statistics were used to calculate relative and absolute frequencies. Exact binominal 95% confidence intervals (CIs) were established for proportions. The chi-square with Yates correction was used to compare proportions with a *p* value < 0.05 regarded as statistically significant. Analyses were performed using Epitools-Epidemiological Calculators (https://epitools.ausvet.com.au/).

## Results

Out of 201 examined dogs, 134 (66.7%; 95% CI = 59.9–72.8%) tested serologically or molecularly positive to at least one vector-borne pathogen. Particularly, *Ehrlichia* spp. was the most frequently detected by serology in 115 (57.2%; 95% CI = 50.3–63.9%), followed by *Anaplasma* spp. 17 (8.5%; 95% CI = 5.3–13.1%), *Leishmania* spp. 17 (8.5; 95% CI = 5.3–13.1%) and *D. immitis* 11 (5.5%; 95% CI = 3.1–9.5%). In addition, simultaneous positivity was observed in 16.9% (*n* = 34) of dogs, mostly by *Ehrlichia* spp. with other pathogens (Table 2). Only one female dog was seropositive for *B. burgdorferi* s.l. with co-positivity to *Ehrlichia* spp. Data on sex, dog category, overnight stay and the presence of ectoparasites are reported in Table 3**,** along with the percentage of dogs positive for each pathogen.

**Table 2:** Simultaneous positivity by canine vector-borne pathogens in Brazil

Pathogen	Prevalence % (*n*)	95% CI
*Ehrlichia* spp. + *Anaplasma* spp.	6.5% (13)	3.8–10.7
*Ehrlichia* spp. + *Leishmania* spp.	5.0% (10)	2.7–8.9
*Ehrlichia* spp. + *D. immitis*	3.0% (6)	1.4–6.4
*Ehrlichia* spp. + *D. immitis* + *Anaplasma* spp.	1.0% (2)	0.3–3.6
*Ehrlichia* spp. + *Borrelia burgdorferi*	0.5% (1)	0.1–2.8
*Ehrlichia* spp. + *Anaplasma* spp. + *Leishmania* spp.	0.5% (1)	0.1–2.8
*D. immitis* + *Leishmania* spp.	0.5% (1)	0.1–2.8

**Table 3:** Number and percentage of dogs seropositive to *Leishmania* spp., *Ehrlichia* spp., *Anaplasma* spp. and *Dirofilaria immitis,* according to their sex, mobility, overnight stay and presence of ectoparasites

Variables	*Leishmania* spp. n/N (%; 95% CI)	χ^2^; *p* value	*Ehrlichia* spp. n/N (%; 95% CI)	χ^2^; *p* value	*Anaplasma* spp. n/N (%; 95% CI)	χ^2^; *p* value	*Dirofilaria immitis* n/N (%; 95% CI)	χ^2^; *p* value
Sex								
Male	8/96 (8.3%; 4.3–15.6)	0.0373; *p* = 0.85	48/96 (50.0%; 40.2–259.8)	3.363; *p* = 0.66	6/96 (6.2%; 2.9–13.0)	0.6754 *p* = 4.11	4/96 (4.2%; 1.6–10.2)	0.219; *p* = 6.39
Female	9/105 (8.6% 4.6–15.5)		67/105 (63.8%; 54.3–72.4)		11/105 (10.5%; 6.0–17.8)		7/105 (6.7%; 3.3–13.1)	
Mobility								
Semi-restricted	17/169 (10.1%; 6.4–15.5)	NP	101/169 (59.8%; 52.2–66.9)	2.2022; *p* = 1.37	13/169 (7.7%; 4.6–12.7)	0.3023; *p* = 5.82	11/169 (6.6%; 3.7–11.4)	NP
Unrestricted	0/32 (0)		14/32 (43.8%; 28.2–60.7)		4/32 (12.5%; 5.0–28.1)		0/32 (0)	
Overnight stay								
Peridomicile	17/167 (10.2%; 6.0–15.7)	NP	101/167 (60.5%; 52.9–67.6)	3.5372; *p* = 0.59	13/167 (7.8%; 4.6–12.9)	0.1782; *p* = 6.72	11/167 (6.6%; 3.7–11.4)	NP
Intradomicile	0/34 (0)		14/34 (41.2%; 26.4–57.8)		4/34 (11.8%; 4.7–26.6)		0/34 (0)	
Ectoparasites								
Yes	7/60 (11.7%; 5.8–22.2)	0.6235; *p* = 4.29	41/60 (68.3%; 55.8–78.7)	3.6968; *p* = 0.54	6/60 (10.0%; 4.7–20.1)	0.0555; *p* = 8.13	8/60 (13.3%; 6.9–24.2)	8.165; *p* = 0.04
Not	10/141 (7.1%; 3.9–12-6)		74/141 (52.5%; 44.3–60.5)		11/141 (7.8% 4.4–13.4)		3/141 (2.1%; 0.7–6.1)	

CI, confidence interval; n, number of dogs positive; N, number total of dogs. χ^2^ = chi-square. Significant at *p* < 0.05. NP indicates

data not possible to calculate

At the molecular analysis, the dog blood samples showed PCR-positive results for *Babesia* spp./*Hepatozoon* spp. in 21 (10.4%; 95% CI = 6.9–15.4%), Anaplasmataceae in 10 (5.0%; 95% CI 2.7–8.9%) and *Rickettsia* spp. in 6 (3.0%; 95% CI 1.4–6.4%). All samples were PCR-negative for the presence *B. burgdorferi* s.l., and for the rickettsial *ompA* gene.

According to BLAST analysis, two samples positive for the 18S rRNA gene revealed 100% nucleotide identity with *B. vogeli* (accession number: MN700646), and 19 samples presented 97.2–100% nucleotide identity with *H. canis* (accession numbers: MK673842; MK645969; MN791089; MK673839). For the 16S rRNA gene sequences, two samples revealed 100% nucleotide identity with *A. platys* (accession numbers: MN922611; MN994338; MN922609; MN922608) and eight samples revealed 99.1–100% nucleotide identity with *E. canis* (accession numbers: MN922610; MN227484; MN227484; MK507008). A single *A. platys* positive sample was also reagent at serological examination, whereas five *E. canis* positive samples were detected at both molecular and serological tests.

Finally, four samples positive for the *gltA* gene had 100% nucleotide identity with *R. felis* sequences (accession numbers: MN817141; MN817140; MT499363).

Sequences herein obtained were submitted to GenBank under the accession numbers: OP084684 for *Babesia vogeli*; OP084681 to OP084683 for *Hepatozoon canis;* OP082323 for *Anaplasma* spp.; OP082324 to OP082328 for *Ehrlichia* spp.; and OP099835 for *Rickettsia felis.*

## Discussion

Data presented herein indicated a high overall level of exposure to vector-borne pathogens in dogs from the *Quilombola* community in northeastern Brazil. In particular, over 50% of the dogs were seropositive to *Ehrlichia* spp., which is in line with previous studies conducted in the same region (Figueredo et al. 2017; Dantas-Torres et al. 2020). This may be partly explained by the high prevalence of the brown dog tick on dogs in the study area (Santos et al. 2017; 2018). The seropositivity to other pathogens (i.e., *Anaplasma* spp., *Leishmania* spp. and *D. immitis*) was lower as compared with a recent study conducted in an urban area of Pernambuco (Dantas-Torres et al. 2020). Differences may be related to many eco-epidemiological factors, including the level of exposure to the vectors and climatic factors. For example, in the same area a low occurrence and diversity in sand fly species throughout a one-year sampling period has been demonstrated, with *Lutzomyia evandroi* being the only species identified (Ubirajara Filho et al. 2020).

Among the vector-borne protozoa, *H. canis* was the most frequently detected molecularly (i.e., *n* = 19 dogs were infected by *H. canis*, while only two dogs by *B. vogeli*). This may be due to the shorter time of circulation of *B. vogeli* in the blood of infected animals. Epidemiological data from previous studies demonstrated that *B. vogeli* is highly prevalent in Brazil, with up to 8.2% molecularly positive dogs in Pernambuco (Ramos et al. 2010; Dantas-Torres et al. 2021), 8.0% in São Paulo (O’Dwyer et al. 2009), 10.0% in Paraíba (Rotondano et al. 2015), 15.0% in Ceará (Fonsêca et al. 2022), 30.6% in Minas Gerais (Barbosa et al. 2020) and 14.1% in Rio de Janeiro (Paulino et al. 2018; Camilo et al. 2021). Conversely, *H. canis* has been reported with lower prevalence (i.e., 0.4% to 5.4%) in Northeastern Brazil (Ramos et al. 2010; Dantas-Torres et al. 2021), differently from the Southeastern region, in which higher prevalence values (i.e., 58.7–79.2%) were reported (Miranda et al. 2014; Spolidorio et al. 2009).

The simultaneous detection of *Ehrlichia* spp. and *Anaplasma* spp. (6.5%) at PCR and serological tests agrees with previous reports in Pernambuco state in which both pathogens were the most frequently diagnosed molecularly (Ramos et al. 2010). The above picture is probably due to the high abundance of brown dog ticks, which may transmit multiple pathogens simultaneously (Shaw et al. 2001). Similarly, the positivity to both *Ehrlichia* spp. and *Leishmania* spp. is of importance due to the implications those pathogens may have in the pathogenesis of CVBD, mainly their clinical presentation, and response to therapy (de Caprariis et al. 2011; Attipa et al. 2018). For example, a study on the experimental infection of *E. canis* and *A. platys* in dogs demonstrated that these pathogens cause various changes in pathophysiological parameters (i.e., more pronounced anemia and thrombocytopenia). Therefore, raising awareness of the risks of simultaneous positivity when animals are exposed to multiple tick-borne pathogens (Gaunt et al. 2010).

*Rickettsia felis*, a zoonotic rickettsia belonging to the SFG, previously described infecting human patients from Kenya (Richards et al. 2010) and Serbia (Banović et al. 2021), was molecularly detected for the first time in the dogs herein assessed. This pathogen was also detected in fleas (*Ctenocephalides felis*) and ticks (*Dermacentor nitens*) collected from dogs and horses from the same study area (Oliveira et al. 2020). *Rickettsia felis* is widespread in Brazil, and its distribution overlaps that of *C. felis*, its main biological vector (Horta et al. 2014). This finding is particularly important from a One Health perspective, considering that the local circulation of zoonotic pathogens may represent an eminent risk for the *Quilombola* community.

## Conclusion

Data herein presented demonstrated that different pathogens (i.e., *B. vogeli, A. platys, E. canis*, *H. canis* and *R. felis*) are prevalent in dogs from the studied *Quilombola* community in northeastern, Brazil. Considering the occurrence of zoonotic pathogens in dogs and their close relationship with humans living in these communities, increased awareness and effective control strategies are advocated for minimizing the risk of infection in animals and humans from *Quilombola* communities, where the access to veterinary services is generally limited.

## Data Availability

The authors declare that data supporting the findings of this study are available within the article.
